# Assessment of the Association between In Vivo Corneal Morphogeometrical Changes and Keratoconus Eyes with Severe Visual Limitation

**DOI:** 10.1155/2019/8731626

**Published:** 2019-09-22

**Authors:** J. S. Velázquez, F. Cavas, J. Alió del Barrio, D. G. Fernández-Pacheco, J. Alió

**Affiliations:** ^1^Department of Structures, Construction and Graphic Expression, Technical University of Cartagena, 30202 Cartagena, Spain; ^2^Keratoconus Unit of Vissum Corporation Alicante, 03016 Alicante, Spain; ^3^Department of Ophthalmology, Miguel Hernández University of Elche, 03202 Alicante, Spain

## Abstract

Assessing changes suffered by the cornea as keratoconus progresses has proven to be vital for this disease diagnosis and treatment. This study determines the corneal biometric profile in eyes considered as affected by keratoconus (KC) showing severe visual limitation, by means of in vivo 3D modelling techniques. This observational case series study evaluated new objective indices in 50 healthy and 30 KC corneas, following a validated protocol created by our research group, which has been previously used for diagnosis and characterization of KC in asymptomatic (preclinical) and mild visually impaired eyes. Results show a statistically significant reduction of corneal volume and an increase of total corneal area in the severe KC group, being anterior and posterior corneal surfaces minimum thickness points the best correlated parameters, although with no discrimination between groups. Receiving operator curves were used to determine sensitivity and specificity of selected indices, being anterior and posterior apex deviations the ones which reached the highest area under the curve, both with very high sensitivity (96.7% and 90%, respectively) and specificity (94.0% and 99.9%, respectively). The results suggest that once severe visual loss appears, anterior corneal topography should be considered for a more accurate diagnosis of clinical KC, being anterior apex deviation the key metric discriminant. This study can be a useful tool for KC classification, helping doctors in diagnosing severe cases of the disease, and can help to characterize corneal changes that appear when severe KC is developed and how they relate with vision deterioration.

## 1. Introduction

Keratoconus (KC) is a bilateral noninflammatory corneal ectasia with a prevalence of 54.5 per 100,000, which is characterized by a stromal thinning that makes the cornea acquire a conical shape, leading to mild to marked visual impairment [1].

The geometric decompensation that causes the conical shape is localized mainly in the temporal lower quadrant of the mean peripheral region [2] due to a loss of tenacity that the corneal structure suffer by a reorientation of its anatomophysiology [3]. In addition, this morphologic decompensation inducts an increase of the high-order optical aberrations [4], showing the patients high values of irregular astigmatism and presenting as their main refractive sign the impossibility of a complete optical compensation of their ametropia by spherocylindrical lenses. Consequently, their corrected visual acuity will be diminished with respect to patients without corneal pathology [5].

There are many classifications in the scientific literature about the degree of severity of keratoconus [6]; however, it is difficult in clinical practice to handle the multiple indices in which these classifications are based, for a proper evaluation of the disease progression. Also, some of these classifications present some singularities, such as Keratoconus Severity Scores [2], which does not consider pachymetry, or the Amsler–Krumeich classification, which does not take into account that patients, depending on their manifest refraction, may show important refractive fluctuations caused by the corneal multifocality that generates the corneal shape [6]. Besides that, from an optical point of view, patients show a certainly deteriorated spectacle-corrected visual acuity during the disease development, in a way that their visual performance worsens with the progression of the severity degree of keratoconus. Following this criterion, a classification of the stages of the disease has been developed [5, 7], depending on the corrected distance visual acuity (CDVA).

Furthermore, our research group has developed a three-dimensional (3D) virtual model of the cornea by means of computational geometry [8]. These models have been validated for the diagnosis of keratoconus basing on geometric parameters of volume [9], to predict the response to refractive surgeries [10, 11] or the response to the intrastromal ring segment implantation in corneas with keratoconus [12], to analyse nonsurgical corneal modifications, such as applanation tonometry for intraocular pressure measurement [13], or to analyse the behaviour of corneal tissue properties in different scenarios [14]. However, to our knowledge the virtual model has not been used to define the biometric profile in keratoconus eyes with severe visual limitation.

**Figure 1:**
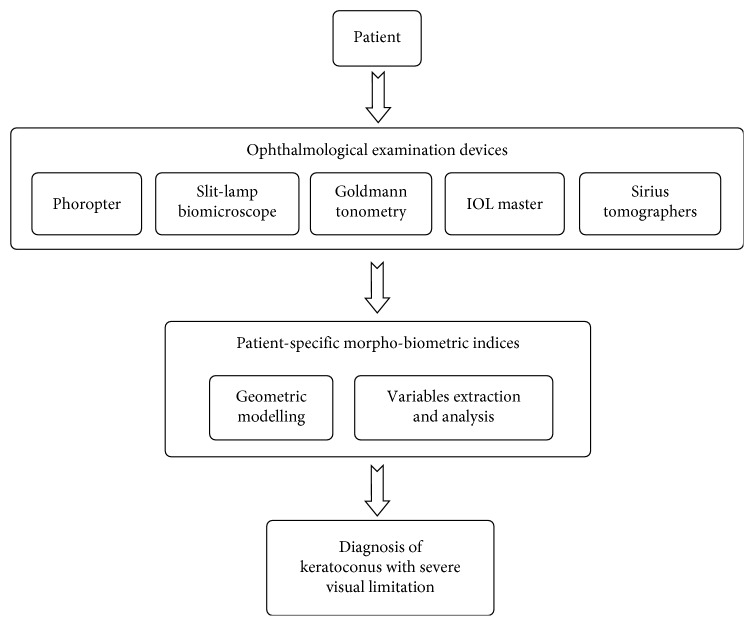
Use of patient-specific morpho-biometric indices for diagnosis of keratoconus with severe visual limitation.

Therefore, developing and validating new methods for the characterization of the changes suffered by the cornea in severe KC cases is important to help attaining a better clinical management of it and avoiding the possibility of irreversible vision losses. Thus, the purpose of this work is to evaluate the potential value of a virtual 3D model for the diagnostic of corneas affected by severe KC, conceiving cornea as a 3D refractive structure. To do so, we have based on the characterization of its biometric profile (Figure 1) by means of morpho-biometric indices that register the optic-geometric decompensation that takes place during this phase of the disease, as well as we have quantified the existence of correlations between these indices.

## 2. Materials and Methods

### 2.1. Participants

This observational case series study evaluated 80 corneas of 80 patients (selected at random to avoid interference) structured in two groups: a normal group (healthy corneas), which included 50 subjects presenting no ocular pathology (37.79 ± 14.76 years), and a second group, composed of 30 patients diagnosed with severe KC (31.63 ± 7.39 years). The classification protocol for normal or severe KC cases was run according to reported state of the art clinical and topographic evaluations [15]. Just one eye of each patient was selected at random, according to a software-generated dichotomic random number sequence (0,1), seeking to elude any possible correlation that might exist between both eyes of the same patient [9].

All patients were selected according to the RETICS grading [7]. To be included in the study, patients should have been diagnosed as Grade IV keratoconus (severe visual limitation, 0.2 < CDVA ≤ 0.4 in decimal scale or 6/30 < CDVA ≤ 6/15 in Snellen chart), focal central/paracentral steepening and corneal thinning visible in corneal tomography, 3 mm (inferior-superior) I-S mean keratometric difference >1.5 D, and asymmetric bow tie with or without skewed radial axes over 21 degrees. Patients who had undergone any previous ocular surgical procedure, suffering from any irritation of the ocular surface, with signs of significant dry eye, or who wore contact lenses in the precedent four weeks to their first visit were excluded from the study [16].

Vissum Instituto Oftalmologico, Alicante, Spain (Vissum), was the place in which these evaluations took place where the patients were satisfactorily informed about the study and signed freely their will to participate. The study was endorsed by the hospital's Clinical Research Ethics Committee, according to the ethical guidelines dictated in the Declaration of Helsinki (Seventh revision, October 2013, Fortaleza, Brazil).

The data used for this investigation were included in the official database “Iberia” of keratoconus cases created for the purpose of multicentre study of keratoconus in the National Network for Clinical Research in Ophthalmology RETICS-OFTARED.

### 2.2. Examination Protocol

**Figure 2:**
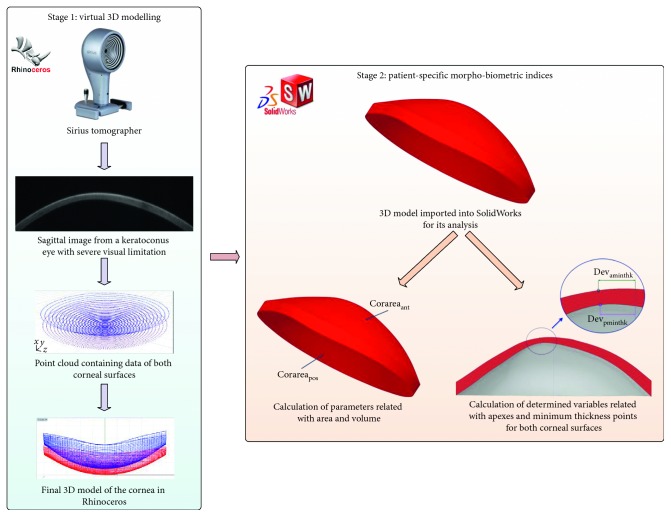
Protocol followed for the creation of the 3D virtual model and its later analysis.

All subjects selected for this study were examined using Sirius System® (CSO, Florence, Italy), and following a validated protocol previously created by our research group, which has been thoroughly described in preceding studies [8, 9]. This protocol comprises two stages: first, a 3D virtual modelling and then geometric characterization (Figure 2), and it has proved itself successful when used for diagnosis and characterization of KC in asymptomatic (preclinical) and mild visually impaired eyes [17, 18].

The final output of this protocol after its application, is a patient-specific 3D virtual model of the cornea, which is then analysed to find several morpho-biometric indices (Figure 2). These indices studied herein, along with their characteristics, have been previously described in [19] and are summarized in Table 1, but are used for the first time to study KC eyes with severe visual impairment. In this work, the surface finally generated with Rhinoceros software was distorted looking for the minimisation of the nominal distance between the points in the space and the surface itself. This distance was ultimately estimated by the software, showing a mean value for its error of 4.370 × 10^−16^ ± 3.67 × 10^−16^ mm (mean ± standard deviation).

### 2.3. Statistical Analysis

Both Kolmogorov–Smirnov test and Shapiro–Wilk test were run to check data normality. According to these tests and thereafter, a Student's *t*-test was used for normally distributed samples, while Mann –Whitney–Wilcoxon U test was chosen for not-normally distributed ones. Correlation between parameters was assessed by means of Pearson coefficient (for normally distributed data) or Spearman's coefficient (not normally distributed). A significance level of 0.05 was fixed for *p*-values in all statistical tests. Receiver operating characteristic (ROC) curves were used to determine which parameters could be useful in terms of characterization of diseased corneas, and optimal cutoffs were established using Youden's J index, basing on sensitivity and specificity values [20, 21]. Graphpad Prism V 6 (GraphPad Software, La Jolla, USA) and IBM SPSS V 23.0 software (SPSS, Chicago, USA) were used to make all the analyses.

## 3. Results

Most of the modelled morpho-biometric indices showed statistically significant differences when comparing healthy and severe KC corneas, as shown in Table 2 below.

### 3.1. ROC Analysis

**Figure 3:**
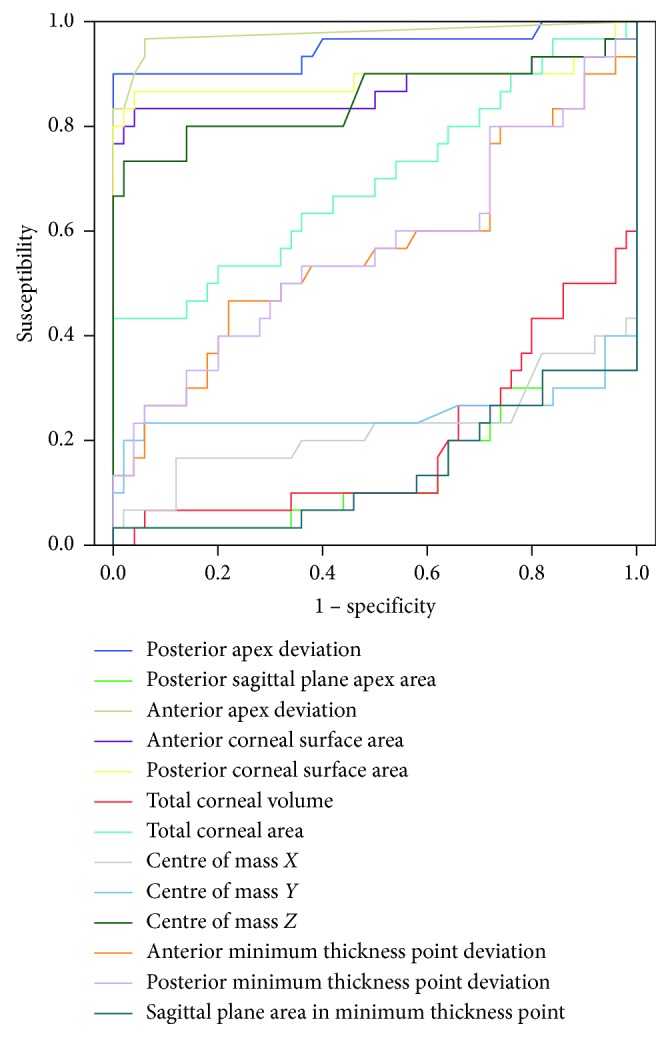
Curves for modelled indices detecting severe KC.

The predictive value of the modelled indices was established by an ROC analysis (Figure 3). Five morpho-biometric indices were identified with an area under the ROC (AUROC) above 0.85 (Table 3).

Table 4 summarizes all significant correlations between the modelled biometric parameters for the severe KC group. Correlation coefficients between parameters for the normal group have not been included, as their mutual relations have already been addressed in a previous study [18].

## 4. Discussion

From a visual point of view, patients of keratoconus disease show a deteriorated spectacle-corrected visual acuity, in such a way that when the disease progresses towards a higher degree of severity, the visual performance of patients gets worse. Thus, it is of great interest evaluating in a jointly manner both the geometric decompensation that takes place in corneal structure due to its structural weakening and the level of visual limitation that patients show during the disease progression. In this work, we analysed the biometric profile of the cornea conceived as a 3D refractive structure for advanced degrees of keratoconus, whose patients present a severe degree of visual limitation, according to the RETICS classification [7].

The volumetric morpho-biometric indices showed a statistically significant reduced total corneal volume in the severe KC group when compared with the healthy eyes group, which is in line with the corneal thinning described by some authors as the disease progresses [16, 22, 23] due to a loss of tenacity in the corneal structure [13, 24]. These results are consistent with the ones reported by previous studies, which have evaluated the same anatomic index with devices based on Scheimpflug technology [23, 25] and similar to the ones reported in previous studies for advanced degrees of keratoconus [9]. Also, some significant positive correlations between corneal volume and sagittal plane apex area of the posterior surface (*R*^2^ = 0.919, *p* ≤ 0.001), and with sagittal plane area at minimum thickness point (*R*^2^ = 0.931, *p* ≤ 0.001) were found, so that when the volume diminishes by the loss of structural resistance that takes place for the progression of the disease, the sagittal areas (Splarea_papex_/Splarea_minthk_) also diminish as the corneal curvature increases. This agrees with the findings of significant lower volumes associated with pachymetric reductions in grade II and higher keratoconus eyes [23].

In addition, regarding the morpho-biometric indices of the anterior and posterior surface areas, these present a statistically significant augmentation, being the greater the area of the posterior surface than the one of the anterior surface, which can be explained by the tendency of the cornea to retain a conical-shaped architecture in the advanced stages of the disease, in which the relation between both surfaces is modified by a higher increase of the posterior surface curvature than the one in the anterior surface, motivated by the biomechanical weakening that cornea manifests for a reorientation of its anatomophysiology [26]. A tendency in this line of structural weakening has been reported in previous studies [13, 26, 27]. Vega-Estrada et al. [7] observed a significant increase in dioptric power of the posterior corneal surface with respect to the anterior in advanced phases of the disease. According to these findings, corneal multifocality that produces the conical shape that locally acquires corneal surface, drives to a worsening in patient's visual performance. Besides, anterior, posterior, and total corneal surface areas show a strong positive correlation between them, and their values increase in the severe KC group, which can be explained by the fact that structural weakening caused by the presence of fewer collagen fibres in each lamella leads to a severe local protrusion that increases the corneal surface [2] by the effect of the intraocular pressure over a structurally weakened biomechanical architecture. These results are coincident with the ones presented in other studies [16, 22, 28].

The anterior and posterior corneal surface minimum thickness point deviations show the strongest correlation between them (*R*^2^ = 0.995, *p* ≤ 0.001). However, these are the only parameters that show no statistical difference for group discrimination (*p*=0.271 and *p*=0.229, respectively). This addresses the relationship between both corneal surface curvatures for keratoconus eyes, as it has already been made in previous studies for both diseased and normal eyes [16, 28], but also that in the case of severe visual limitation, this deviation varies so greatly among individuals that discrimination is impossible.

Anterior apex deviation and posterior apex deviation increase for the severe KC group. This displacement of the optical axis has been described as one of the signs of the later stages of the disease [1]. This is in concordance with some authors' findings [23] that suggest a strong correlation between apex deviation and pachymetric progression index of the front and back elevations with CDVA of the analysed patients. Also, posterior apex deviation presents an important variability with respect to the anterior apex deviation, this is motivated by the tendency of the aspheric profile to reproduce the cornea's physiologic prolatism in advanced phases of the disease [16, 28]. It can even be observed a paraboloid type geometry in virtual 3D models, a fact that can be relevant to explain the asymmetry that shows the posterior corneal surface in advanced keratoconus. This variability has been reported in previous studies [15, 29, 30], being this one the first in which posterior surface asymmetry is quantified for keratoconus eyes with severe visual limitation. Besides, this geometric tendency is correlated with the increase of optical aberrations in advanced degrees of keratoconus [5, 22, 31].

Results also suggest a strong correlation between centre of mass Z and anterior, posterior, and total corneal surfaces (*R*^2^ = 0.925, *p* ≤ 0.001; *R*^2^ = 0.874, *p* ≤ 0.001; *R*^2^ = 0.873, *p* ≤ 0.001, respectively) as well as a displacement of the centre of mass along the Z-positive axis, which is logical, as the loss of corneal volume that steeps the surfaces, should also force the centre of mass to move towards the protrusion, as a consequence of the corresponding displacement of both apex points in the Z-coordinate. This is in line with the findings of some authors [32, 33], which found that the mean maximum anterior and posterior corneal elevations were higher in eyes with subclinical or clinical keratoconus.

Regarding the ROC analysis, the anterior apex deviation reached the highest area under the curve (AUC, 0.977) with very high sensitivity (96.7%) and specificity (94.0%) due to the fact that the apex is the maximum curvature point of the corneal surface. Moreover, the posterior apex deviation also shows high discrimination capability (AUC, 0.948) with very good sensitivity (90%) and excellent specificity (99.9%). Anterior and posterior corneal surfaces and centre of mass show high AUC, but although their specificity is high, their sensitivity is not as good as the one for the deviation of the apices.

In conclusion, the analysis of corneal biometric parameters using patient-specific 3D modelling has ascertained statistically significant differences between normal and KC eyes with severe visual limitation. This computer-assisted custom approach has determined several indices that successfully characterize the profile of keratoconic eyes affected by severe visual limitation. Therefore, this analysis has proven to be a useful tool for KC classification, helping doctors to achieve reliable diagnoses in severe cases of the disease, as well as it has helped to better characterize corneal changes that take place when severe KC is developed and how they relate with vision deterioration.

## Figures and Tables

**Table 1:** Patient-specific morpho-biometric indices analysed in the study [19].

Morpho-biometric parameters	Description
Total corneal volume (Vol_tot_) (mm^3^)	Volume limited by front, back, and peripheral surfaces of the solid model generated

Anterior/posterior corneal surface area (Corarea_ant_/Corarea_pos_) (mm^2^)	Area of the front/exterior and rear/interior surfaces

Total corneal surface area (Corarea_tot_) (mm^2^)	Sum of anterior, posterior, and perimeter corneal surface areas of the solid model generated

Sagittal plane apex area (Splarea_papex_) (mm^2^)	Area of the cornea within the sagittal plane passing through the optical axis and the highest point (apex) of the posterior corneal surface

Anterior and posterior apex deviation (Dev_aapex_/Dev_papex_) (mm)	Average distance from the optical axis to the highest point (apex) of the anterior/posterior corneal surfaces

Sagittal plane area in minimum thickness point (Splarea_minthk_) (mm^2^)	Area of the cornea within the sagittal plane passing through the optical axis and the minimum thickness point (maximum curvature) of the posterior corneal surface

Anterior and posterior minimum thickness point deviation (Dev_aminthk_/Dev_pminthk_) (mm)	Average distance in the *XY* plane from the optical axis to the minimum thickness points (maximum curvature) of the anterior/posterior corneal surfaces

Centre of mass *X*, *Y*, *Z* (COM_*X*_, COM_*Y*_, COM_*Z*_) (mm)	Centre of mass coordinates *X*, *Y*, *Z* of the solid

**Table 2:** Descriptive values and differences in the modelled morpho-biometric indices among the normal and severe KC groups.

Morpho-biometric indices	Normal group (*n* = 50)				Severe KC group (*n* = 30)					
	Mean	SD	Min	Max	Mean	SD	Min	Max	*z*	*p*
Vol_tot_ (mm³)	25.72	1.53	23.23	29.07	23.74	1.85	20.17	28.40	5.18	≤0.01
Corarea_ant_ (mm^2^)	43.08	0.14	42.77	43.33	43.80	0.59	42.66	45.11	−6.56	≤0.01
Corarea_pos_ (mm^2^)	44.24	0.26	43.53	44.71	45.44	0.95	43.78	47.97	−6.78	≤0.01
Corarea_tot_ (mm^2^)	103.89	1.12	100.73	105.66	105.20	2.13	102.35	112.68	−3.14	≤0.01
Splarea_papex_ (mm^2^)	4.32	0.26	3.93	4.87	3.86	0.40	2.96	5.02	6.33	≤0.01
Splarea_minthk_ (mm^2^)	4.31	0.26	3.92	4.86	3.84	0.40	2.97	5.03	6.41	≤0.01
Dev_aapex_ (mm)	0.00	0.00	0.00	0.00	0.03	0.02	0.00	0.06	−8.26	≤0.01
Dev_papex_ (mm)	0.07	0.02	0.04	0.09	0.26	0.12	0.05	0.59	−6.68	≤0.01
COM_X_ (mm)	0.04	0.02	0.01	0.09	−0.01	0.06	−0.20	0.12	4.23	≤0.01
COM_Y_ (mm)	0.03	0.02	0.00	0.08	−0.01	0.09	−0.13	0.33	2.57	0.02
COM_Z_ (mm)	0.77	0.02	0.71	0.81	0.85	0.08	0.70	1.06	−5.56	≤0.01
Dev_aminthk_ (mm)	0.83	0.21	0.44	1.27	0.91	0.36	0.16	1.68	−1.12	0.27
Dev_pminthk_ (mm)	0.76	0.20	0.38	1.24	0.85	0.34	0.10	1.60	−1.23	0.23

SD: standard deviation; *P*: statistical test; *z*: *z*-score.

**Table 3:** The area under the ROC results.

Morpho-biometric indices	AUROC	Sensitivity	Specificity	Cutoff value
Corarea_ant_	0.874	83.3	96.0	≥43.275 mm^2^
Corarea_pos_	0.890	86.7	96.0	≥43.160 mm^2^
Dev_aapex_	0.977	96.7	94.0	≥0.001 mm
Dev_papex_	0.948	90.0	99.9	≥0.098 mm
COM_*Z*_	0.852	73.3	98.0	≥0.809 mm

**Table 4:** The significant correlation coefficient values for the modelled variables in the severe KC group.

Measurement correlation	Severe KC group (*n* = 30)	
	Correlation coefficient	*p* value
Splarea_papex_/Vol_tot_	0.919	≤0.001
Corarea_ant_/Corarea_pos_	0.917	≤0.001
Corarea_ant_/Corarea_tot_	0.835	≤0.001
Corarea_pos_/Corarea_tot_	0.899	≤0.001
Vol_tot_/Corarea_tot_	0.439	0.015
COM_*Y*_/Splarea_papex_	0.448	0.013
COM_*Z*_/Dev_aapex_	0.366	0.046
COM_*Z*_/Corarea_ant_	0.925	≤0.001
COM_*Z*_/Corarea_pos_	0.874	≤0.001
COM_*Z*_/Corarea_tot_	0.873	≤0.001
Dev_aminthk_/Dev_papex_	0.597	≤0.001
Dev_pminthk_/Dev_papex_	0.611	≤0.001
Splarea_minthk_/Splarea_papex_	0.989	≤0.001
Vol_tot_/Splarea_minthk_	0.931	≤0.001
COM_*X*_/COM_*Y*_	−0.375	0.041
COM_*X*_/Dev_aminthk_	−0.429	0.018
COM_*X*_/Dev_pminthk_	−0.476	0.008
COM_*Y*_/Splarea_minthk_	−0.403	0.027
Dev_aapex_/Dev_papex_	0.995	≤0.001

## Data Availability

The data used to support the findings of this study were supplied by “Iberia” under license and so cannot be made freely available. Requests for access to these data should be made to Dr. Jorge Alió, jlalio@vissum.com, National Network for Clinical Research in Ophthalmology RETICS-OFTARED.
